# The fertility-sparing treatment and outcome of epithelioid trophoblastic tumor isolated to lung: a case report and review literature

**DOI:** 10.3389/fonc.2024.1337213

**Published:** 2024-03-14

**Authors:** Zengshu Huang, Yingjuan Yu, Darong Wen, Nan Wang, Liping Zeng

**Affiliations:** ^1^ Center of Obstetrics and Gynecology, Peking University Shenzhen Hospital, Shenzhen, China; ^2^ Institute of Obstetrics and Gynecology, Shenzhen Peking University - The Hong Kong University of Science and Technology (PKU-HKUST) Medical Center, Shenzhen, China; ^3^ Shenzhen Key Laboratory on Technology for Early Diagnosis of Major Gynecologic Diseases, Shenzhen, China

**Keywords:** epithelioid trophoblastic tumor (ETT), isolated pulmonary metastasis, fertility-sparing surgery, case report, literature review

## Abstract

**Background:**

Epithelioid trophoblastic tumor (ETT) is the rarest gestational trophoblastic tumor, with poor response to chemotherapy. Hysterectomy, as the cornerstone therapy for early ETT, is particularly challenging in reproductive-age women who often have a strong desire for fertility preservation. The management of extra-uterine ETT could be even more complicated and inconsistent. Here we reported a case of isolated ETT lesions in lungs managed with thoracic surgery without hysterectomy.

**Case presentation:**

A 32-year-old woman presented with amenorrhea for 2 months. Her serum β- human chorionic gonadotropin (hCG) levels fluctuated between 52 and 75 mIU/mL. The patient underwent removal of intrauterine device and suction and curettage, but only proliferative endometrium was found. Methotrexate was given for a provisional diagnosis of ectopic pregnancy of unknown location, while β-hCG had no significant decline. She complained of mild chest pain during the past half year, and the chest computed tomography (CT) result showed two mixed ground-glass nodules of 24 mm × 14.2 mm in right upper lobe and 10 mm × 8 mm in the right lower lobe and a thin-walled cavity in the posterior segment of the left lower lobe. Right upper wedge resection and right lower segmentectomy were performed 3 months later. The result of the pathological examination of pulmonary mass indicated an epithelioid trophoblastic tumor. She was diagnosed with ETT at stage III (with right lung metastasis) according to FIGO 2000. Her menstrual cycle recovered within 1 month after the first thoracic surgery. However, β-hCG was elevated again to 9 mIU/mL, and the positron emission tomography/computed tomography (PET/CT) scans revealed the consolidation of the nodule in the left lower lobe which enlarged to about 1.0 cm × 1.7 cm. Her second pulmonary surgery without hysterectomy was conducted. Followed for 12 months for postoperative monitoring, the patient was found to be disease-free with negative results of serial serum β-hCG and chest CT.

**Conclusion:**

Our case highlights the efficacy of fertility-sparing surgery for isolated ETT in lungs. The surgical management of pulmonary isolated ETT could be individualized under long-term supervision. Sporadic reports on the favorable outcome of extra-uterine ETT with fertility-sparing surgery were described in the last decades. The safety of this surgical strategy might be warranted only if enough reliable data is accumulated.

## Introduction

Epithelioid trophoblastic tumor (ETT), a rare subtype of gestational trophoblastic neoplasia (GTN), is derived from chorion laeve-type extravillous intermediate trophoblasts. Lung is the major target organ of distant metastasis (1). A unique clinical phenomenon was observed in a few ETT cases, which presented with isolated lung ETT without a known primary origin in the reproductive system. In addition to the case from our center, there are only 28 reported cases worldwide so far (reviewed literature written in English). Two main hypothesis were proposed to explain the potential pathogenesis of this disease: one is that the primary trophoblastic stem cells may experience further malignant transformation into ETT after being transferred to the lungs during early pregnancy (2) and another one is that the isolated lung lesions may result from the spontaneous regression of uterine ETT at an early stage (3).

Hysterectomy is the standard treatment for patients with ETT confined to the uterus (stage I, according to FIGO 2000 with World Health Organization), which can achieve complete remission (4). Nevertheless, the benefit of supplementary hysterectomy after excision of isolated pulmonary lesion is controversial in patients with isolated lung lesions but no primary lesion in the uterus. These women usually are young and have a strong desire for fertility preservation. The indication and outcome of a fertility-sparing therapeutic regimen for those cases are worthy of further discussion and verification.

Here we reported a case of a child-bearing-period woman with an initial symptom of amenorrhea and chest pain caused by isolated lung mass of ETT. The patient acquired satisfactory outcome after receiving her individualized treatment based on fertility-sparing surgery.

## Case presentation

**Figure 1:**
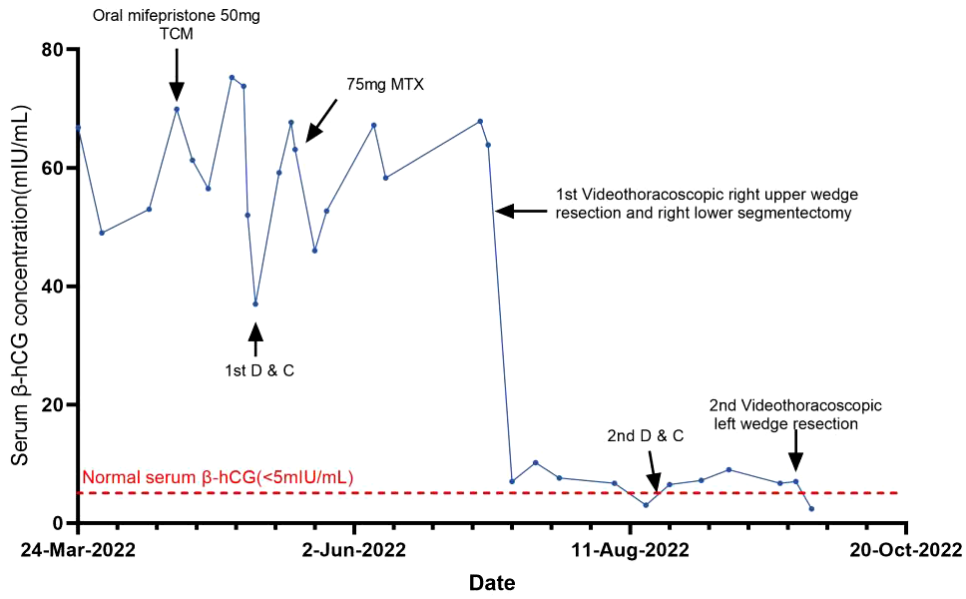
Curve showing serum β-hCG during the diagnosis of ETT and in response to the treatment procedures.

**Figure 2:**
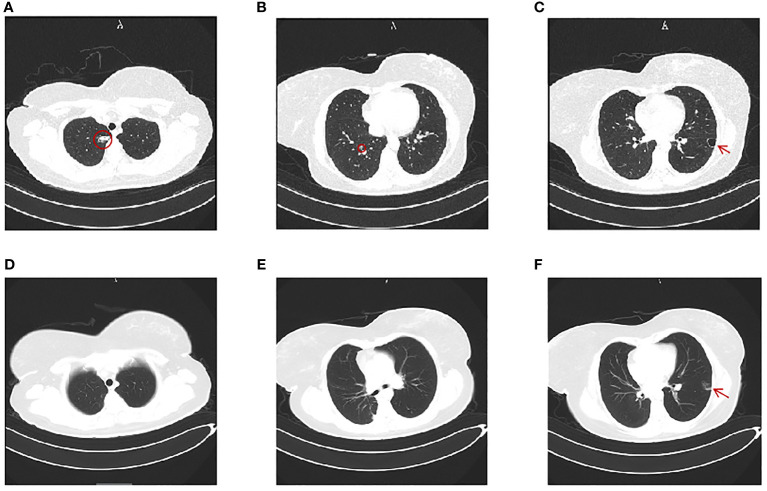
Chest computerized tomography (CT). **(A–C)** Chest CT image before the first video-assisted thoracoscopic right upper wedge resection and right lower segmentectomy. **(A)** A mGGN of 24 mm × 14.2 mm in the apical segment of the upper lobe (red circle). **(B)** A mGGN of 10 mm × 8 mm in the posterior basal segment of the right lung, marked by a red circle. **(C)** A thin-walled cavity in the posterior segment of the left lower lobe (red arrow). **(D–F)** Chest CT image 2 months after the first thoracic surgery. **(D, E)** Postoperative changes of the right lung; no mGGN seen. **(F)** The mGGN in the posterior segment of the left lower lobe was enlarged at 10 mm × 17 mm and suspected as nodular consolidation (red arrow).

A 32-year-old woman (married, gravida 6, para 2) presented to our clinic with cessation of menstruation for 51 days. She reported no nausea, vomiting, vaginal bleeding, abdominal pain, or bloating. The physical examination revealed no abnormality in the vagina and uterus. The serum β-hCG was 66.2 IU/ml (**Figure 1**). The transvaginal ultrasound showed an intrauterine device (IUD) in normal position in the uterine cavity. A woman of reproductive age who presented with amenorrhea, elevated serum β-hCG level, and the absence of an intrauterine embryo but IUD on transvaginal ultrasonography should be primarily suspected of ectopic pregnancy. The patient was treated with oral mifepristone at 50 mg combined with traditional Chinese medicine for ectopic pregnancy once a day (5) for 3 days in all. A few days later, she reported minor vaginal bleeding. The serum β-hCG levels fluctuated between 52.0 and 75.3 mIU/mL within 25 days. Subsequently, she was hospitalized for the removal of IUD and underwent dilation and curettage (D&C). The pathological examination revealed proliferative endometrium with no villi structure. Within 10 days after suction and curettage, the serum β-hCG rose to 63.1 mIU/mL despite a repeated unremarkable transvaginal ultrasonography examination. The patient was suspected with ectopic pregnancy in unknown location and administered with 75 mg of methotrexate (MTX) chemotherapy through muscle injection. However, her serum β-hCG remained at persistently low elevations. During her second gynecological hospitalization, she added the complaint of mild chest pain in the past half year, denying cough or hemoptysis. The chest CT showed a mixed ground-glass nodule (mGGN) (24 mm × 14.2 mm) in the apical segment of the right upper lobe which was considered to be invasive cancer and a mGGN (10 mm × 8 mm) in the posterior segment of the right lower lobe undetermined (**Figure 2**). There was another one thin-wall cavity in the posterior segment of the left lower lobe. We invited a consultation with the thoracic surgeon immediately. Antibiotic was recommended to differentiate from inflammatory pulmonary nodules, and no significant degradation was observed on chest CT scan images at 1 month later. The patient was hospitalized thirdly for video-assisted thoracoscopic surgery (VATS).

**Figure 3:**
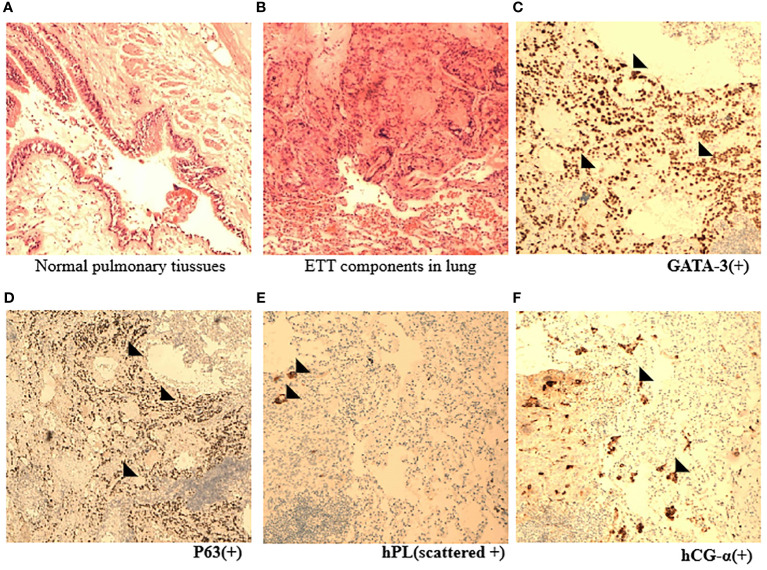
Pathological appearance of ETT. **(A, B)** Representative HE images indicated nests and cords of intermediate epithelioid tumor cells mixed with eosinophilic hyaline-like material in normal lung tissues (×10). **(C–F)** Immunohistochemical staining images showed the expression of specific ETT markers, such as GATA, P63, hPL, and hCG-α (×10) (black arrowhead).

Video-thoracic right upper wedge resection was performed. Intraoperative fast frozen pathology of the resected right upper lobe specimen showed an epithelial-derived tumor consisting of multiple psammoma bodies. Right lower segmentectomy was continued. The fast frozen pathology of the right lower lobe specimen was consistent with that of the right upper lobe. The mitotic count was 20 mitotic figures (MFs)/10 high power fields (HPFs) and the MIB1 proliferative index (Ki-67) was 40%. No tumor metastasis was found in the 11th hilar lymph node. The histology of two resected pulmonary specimens reported an epithelioid trophoblastic tumor (**Figure 3**). The immunohistochemistry analyses showed GATA-3(+), hCG-a(+), hPL(+), P63(+), Ki-67(40%+), CD10(+), Inhibinα(mostly +), CK(pan)(+), Calcitonin(-), TTF-1(-), NapsinA(-), CgA(-), and SYN(-). These findings indicated a pulmonary ETT at stage III presenting as right lung metastasis with unclear primary lesions according to the FIGO 2000.

Her menstruation returned with a sharp drop in serum β-hCG levels, and the mild chest pain was relieved after her thoracoscopic surgery within 1 month. Cranial CT scans were performed, and no metastatic lesions were detected. The patient was very young and expressed strong willingness to preserve her fertility. She refused uterine removal surgery; therefore, we were not able to thoroughly verify the absence of lesions in the primary uterine site through histopathology. We repeated D&C instead of hysterectomy and failed to find intrauterine ETT lesions. Weekly serum β-hCG assessments for surveillance were recommended until three consecutive normal assays, supplemented with monthly β-hCG monitoring for an additional 6 months. However, mildly recurrent elevation of β-hCG was reported 1 week after her first normalization of serum β-hCG. The PET-CT scans showed that the nodule in the left lower lobe was enlarged, and there were more solid components compared with the former chest CT. The lesion was about 1.0 cm × 1.7 cm, measuring the maximum standardized uptake value (SUV max) of 1.8 compared with the average mean SUV from normal lung tissue which was 0.45. Given the recurrence of ETT, she was admitted at our hospital to receive left pulmonary wedge resection. The results of fast frozen biopsy, routine pathological examination, and immunohistochemistry were confirmed to indicate ETT. A pulmonary surgeon agreed with the likelihood of secondary remission for surgery after relapse. In a multidisciplinary meeting, obstetrics and gynecology, thoracic surgery, and pathology experts discussed and shared the final decision for a long-term management involving the fertility-sparing surgery and a critical follow-up plan. At 3 days later, the serum β-hCG was normalized. The patient refused to take the chemotherapy treatment since there were no signs of relapse after having surgery twice and due to fear of adverse reactions such as myelosuppression and hair loss. At the 12-month follow-up after surgical therapy completion, the patient was disease-free with negative β-hCG. The chest CT scans at 3 and 11 months after surgery were performed, and no metastatic lesions were detected. As of the 12-month follow-up, no recurrence has been observed.

## Discussion

The pathogenesis of ETT has not been well explained so far. The DNA sequencing result of new alleles and Y chromosome gene loci from the paternity indicates that ETT originates from fetal tissues (placenta) rather than maternal tissues. ETT may follow any type of a gestational event—mostly after non-hydatidiform pregnancy (including term pregnancy, spontaneous abortion, ectopic pregnancy)—and very few after hydatidiform mole (HM) and invasive mole (6–8).

ETT exhibits similar biological invasiveness but with greater resistance to chemotherapy compared to choriocarcinoma (CCA) due to specific gene mutations related to chemo-resistance (9). Therefore, CCA can effectively be cured through chemotherapy alone, while the primary focus of treatment of ETT is surgery.

The NCCN Guidelines for GTN (2024.V1) just updated the recommendations of different surgery approaches for non-metastatic and metastatic ETT (10). Women with ETT confined to the uterus (stage I) are managed with hysterectomy with salpingectomy (7). Currently, it is not routine to consider ovarian removal, and routine lymph node dissection is considered with large, deeply invasive tumors. Due to the low incidence of ETT, there are yet no consensus on poor prognostic factors. Some researchers included the interval of over 4 years (1) [or over 2 years (7)] from the preceding pregnancy as a risk factor for ETT prognosis besides patient age (≥ 40 years) (11), a high mitotic count (>5–10 MFs/10 HPFs) (11), and the number of metastatic lesions (≥3) (8). Lung metastasis comes to stage III where hysterectomy with salpingectomy and excision of metastatic disease are recommended if feasible (12). However, the favorable outcome of ETT patients with isolated pulmonary lesions resembles that of patients in stage I (8). There were no reported fatalities in 28 cases of isolated pulmonary ETT (**Table 1**). It is suggested that these highly selected patients with isolated chemo-resistant tumor could be managed as clinical stage I by the surgical removal of metastatic ETT lesions (23).

**Table 1:** Clinical features of patients with isolated pulmonary ETT in the literature.

Authors	Patient No.	Age, years	Symptoms	Preceding pregnancy	Interval from last pregnancy, months	Provisional diagnosis	Diagnostic Method	Number of metastases in lungs	Pretreatment hCG, mIU/mL	Surgical resection of pulmonary lesion	Hysterectomy	Preoperative chemotherapy	Postoperative chemotherapy	Initial remission, months	Recurrence	Subsequent treatment	Second remission, months	Follow-up, survival, months
Lei et al. (13)	1	49	None	HM	NA	LC	VATS	1	NA	Thorascopic lower left lobectomy with mediastinal lymphadenectomy	–	–	–	3	–	/	/	3
Urabe S et al. (14)	2	38	Poor physical condition	TPY	NA	NA	VATS	Multiple	80.1	Thorascopic right segmentectomy	–	6 cycles of EMA-CO	+	6	+	Surgical resection of pulmonary lesions	3	9
Shih et al. (3)	3	42	None	TPY	NA	NA	TS	NA	1,300	VATS	–	–	DVLM	Lost	NA	/	/	2
Jia-Wen Li et al. (2)	4	31	None	Subclinical miscarriage	48	NA	VATS	1	168.1	Thorascopic left upper lobe segmentectomy	–	–	3 cycles of EP-EMA	13	–	/	/	13
Lewin SN et al. (15)	5	38	None	TPY	42	Large cell LC	VATS	1	400	Thorascopic right wedge resection and lobectomy	–	–	–	90	–	/	/	90^a^
	6	49	VB	Miscarriage	12	LCA	PTNB, TS	1	2,204	Right lower lobectomy with mediastinal lymph node dissection	+	3.5 cycles of EMA/EP	–	45	–	/	/	45
	7	34	Irregular menses	TPY	24	NA	TS	1	426	Right upper lobe segmentectomy	+	4 cycles of MTX	3 cycles of EMA/EP	22	–	/	/	22
Okereke IC et al. (16)	8	40	VB	NA	NA	NSCLC	PTNB, VATS	1	1,100	Thorascopic lobe lobectomy with mediastinal lymph node dissection	–	–	4 cycles of cisplatin/etoposide	12	–	/	/	12
Kim JY et al. (17)	9	35	Abdominal pain, nausea, and vomiting	NA	NA	SCLC	PTNB, VATS	1	NA	Thorascopic left lower lobectomy with mediastinal lymph node dissection	–	–	–	15	–	/	/	15
Ahn HY et al. (18)	10	26	Delayed and relatively heavy menstruation	Suspected subclinical miscarriage	48	LC	PTNB, VATS	1	NA	Thorascopic right lower lobectomy with mediastinal lymph node dissection	–	–	6 cycles of EMA/CO	9	–	/	/	9
Jingnan Li et al. (12)	11	28	VB	MP	17	GTN	VATS	1	2,021	VATS in right lung	–	5 cycles of TP	2 cycles of EP-EMA + 5 cycles of EP	NA (but remission)	–	/	/	NA
	12	23	None	Abortion	11	Ectopic pregnancy	VATS	1	277		+	–	3 cycles of EP-EMA		–	/	/	
	13	32	VB, cough	TPY	3	LC	VATS	1	194		+	–	3 cycles of EP-EMA		–	/	/	
	14	30	VB	MP	14	GTN	PTNB, VATS	1	144		–	–	3 cycles of EMA-CO		–	/	/	
Liu et al. (8)	15–20	30.5 (average age)	NA	NA	NA	NA	VATS	NA	213 (average)	Pulmonary lesion resection	–	NA	NA	NA (but remission)	–	/	/	30.5 (median)
	21		NA	NA	NA	CCA or IM	VATS			Pulmonary lesion resection	+	+^b^	–	6	+	Pulmonary lesion resection and hysterectomy	NA	
	22									Pulmonary lesion resection	–	+^c^	–	NA (but remission)	–	/	/	
Abrão FC et al. (19)	23	31	Irregular VB	TPY	96	Miscarriage, NSCLC	VATS	1	700	Thorascopic right lower lobectomy and systematic mediastinal lymphadenectomy	–	–	–	12	–	/	/	12
Sobecki-Rausch J et al. (20)	24	28	None	TPY	48	Ectopic pregnancy	VATS	1	50	Thorascopic wedge resection of the right lower lobe	+	–	3 cycles of TP/TE	47	–	/	/	47
Fénichel P et al. (21)	25	29	VB	TPY	48	Ovarian β hCG-secreting germ-cell tumor, NSCLC	PTNB,VATS	1	250	Unilateral ovariectomy, thorascopic left superior lobectomy	–	–	6 cycles of EP-EMA	12	–	/	/	12 (vaginal delivery after a year)
Hamazak et al. (22)	26	47	None	IM	36	Lung metastasis of breast cancer	TS	1	0.7 ng/mL	Thorascopic right upper lung lesion resection	^d^	–	–	24	–	/	/	24
	27	32	None	MP	60	LC	TS	1	NA	Right upper lobectomy	–	–	–	36	–	/	/	36
	28	42	Hemoptysis and cough	NA	NA	LC	TS	1	NA	Left upper lobectomy and lymph node dissection	–	–	+	24	–	/	/	24

*abdominal hysterectomy with left salpingo-oophorectomy 19 months later due to ovarian serous cystadenoma; **hysterectomy and chemotherapy for invasive mole three years ago; # first remission with chemotherapy as initial therapy due to misdiagnosis; ※ not remission with chemotherapy as initial therapy until additional excision of the isolated pulmonary lesion; NA, not available; hCG, human chorionic gonadotropin; VB, vaginal bleeding; TPY, term pregnancy; HM, hydatidiform mole; IM, invasive mole; MP, molar pregnancy; CCA, choriocarcinoma; LC, lung cancer/lung carcinoma; NSCLC, non-small cell lung cancer; GTN, gestational trophoblastic neoplasia; VATS, video-assisted thoracoscopic surgery; TS, thoracic surgery; PTNB, percutaneous transthoracic needle biopsy; EMA, etoposide, methotrexate and actinomycin D; CO, cyclophosphamide, vincristine; EP, etoposide, cisplatin; TP, paclitaxel, cisplatin; TE, docetaxel, epirubicin; MTX, methotrexate; DVLM, dactinomycin, VP16, methotrexate, and leucovorin.

a Abdominal hysterectomy with left salpingo-oophorectomy 19 months later due to ovarian serous cystadenoma.

b First remission with chemotherapy as initial therapy due to misdiagnosis.

c Not remission with chemotherapy as initial therapy until additional excision of the isolated pulmonary lesion.

d Hysterectomy and chemotherapy for invasive mole 3 years ago.

+ positive; - negative; NA not available; /, blank.

In the previously reported 28 cases of isolated pulmonary ETT, operations were performed (2, 8, 12–22) (**Table 1**). Among these cases, 21 cases underwent thoracic surgery without hysterectomy (T-H), while seven cases had the isolated lung lesions removed along with total uterine (T+H) where postoperative pathology revealed a benign disease in uterine. In most of the reported isolated pulmonary ETT cases, uterine is not affected as proven by pathological section after hysterectomy. One case achieved initially partial remission after surgery T-H in unilateral lung but experienced a recurrence of lung lesions 6 months later due to the incomplete removal of tiny metastatic foci in both lungs; a subsequent resection of the lung lesion led to a second partial remission with a slightly elevated β-hCG level (3.8 mIU/mL) (14). Two cases were provisionally diagnosed as CCA or IM and received chemotherapy as the initial treatment (8). One patient achieved the first complete remission by T-H surgery, with the removal of the isolated lung lesion after chemotherapy. The other patient experienced a recurrence and achieved a second remission through the T+H surgery. By statistical analyses of these 29 cases (including our case), we found that complete remission rate [T-H, 95.2% (20/21) vs. T+H, 100% (7/7)], recurrence rate [T-H, 9.5% (2/21) vs. T+H, 0% (7/7)], and overall survival rate [T-H, 100% (22/22) vs. T+H, 100% (7/7)] had no significant difference between the two options (**Table 2**). The necessity of additional hysterectomy is questioned. ETT often occurs in young women aged 15 to 48, with a median age of 32 to 38 (7, 24). The increasing attention of fertility protection prompts a more critical evaluation of the feasibility of uterine preservation by doctors. The key to reducing the risk of relapse with isolated pulmonary ETT seems to be a complete resection of all isolated lesions in the lungs in the first operation rather than the additional hysterectomy.

**Table 2:** Surgery treatment and outcome of patients with isolated pulmonary ETT.

Therapy methods			All patients, *n*	Complete remission, *n*	Recurrence, *n*	Overall survival, *n*
Thoracic surgery	+/-chemotherapy	+	10	8	1	10
		–	6	6	1	6
		Unknown	6	6	0	6
	In total		22	20	2	22
Thoracic surgery + hysterectomy	+/-chemotherapy	+	6	6	0	6
		–	1	1	0	1
	In total		7	7	0	7

It is important to note that there is currently no standardized chemotherapy regimen or recommended number of consolidation chemotherapy cycles for ETT. Whether postoperative β-hCG levels are abnormal or normal, there are quite a few patients who received postoperative adjuvant chemotherapy (2, 12). Some scholars suggested that patients whose postoperative serum β-hCG levels that return to normal immediately have no need for chemotherapy (8). Platinum-based systemic chemotherapy regimens are commonly used, including EMA-EP (etoposide, cisplatin, etoposide, methotrexate, and actinomycin-D) and TP/TE (paclitaxel–cisplatin/paclitaxel–etoposide) (a review of the current management of PSTT and ETT). Regarding adverse actions on the reproductive system of platinum-based chemotherapy, there are no reported cases utilizing the GnRH agonist protocol for ovarian protection before treatment so far. In recent years, immunotherapy, like pembrolizumab (anti-PD-1), has been introduced into GTN, especially unresectable, chemotherapy-resistant GTN cases (25). ETT also indicates high PD-1/PD-L1 gene expression (26, 27). A combination of surgery and immune checkpoint inhibitors could be considered as a salvage option, especially for those of poor reaction to adjuvant chemotherapy.

Given the similarity of the effectiveness between these two surgery options and the less necessity of chemotherapy, it appears that T-H surgery without chemotherapy may be an alternative in these cases of isolated lung ETT lesions. Although unconventional, this concept of fertility-sparing surgery stands out for its unique advantages and simultaneously guarantee a notable degree of safety. The uterine of an isolated pulmonary ETT patient is thought to be better protected during fertility-preservation treatment compared to stage I patients with uterus ETT lesionectomy. The conception and delivery under professional guideline and supervision might be practical after isolated pulmonary ETT disease-free. The treatment is experimental and challenging but truly deserves earnest consideration for the current clinical dilemma. Based on very few available data, we here raised some possible indications for the fertility-sparing surgery of isolated pulmonary ETT: (1) Patients with a pregnancy wish and have all of following conditions, (2) age ≤35 years, (3) the lesion is confined to the lungs without lymph node metastasis, the number of metastatic lesions is ≤3, and the size of the largest tumor is ≤3 cm, (4) interval from index pregnancy is ≤2 years, (5) good compliance and follow-up conditions, and (6) no history of infertility.

Our patient presented with isolated pulmonary ETT lesions without uterine involvement and strongly required for preserving fertility. The assessment by her first onset of ETT included the largest numbers of ETT lesions in the lungs = 3, the largest size <3 cm, and 2 years after the last full-term pregnancy at her age of 32 years. The result of the lung lymph node biopsy was negative. The β-hCG level declined sharply within 1 week of the pulmonary resection, which is usually associated with a good outcome (pulmonary resection in the management of high-risk GTN). Her conditions were roughly qualified with all the indications of a fertility-sparing treatment of ETT. The personal management plan for this ETT patient had been fully discussed by the multi-disciplinary teams including obstetrics and gynecology, thoracic surgery, and pathology. After thoracic surgeries, it is essential to continue the weekly monitoring of serum hCG levels for a consecutive 4-week period and switch to monthly measurements upon three consecutive negative results in the first year. A chest CT scan, including at least one PET/CT scan, is recommended at every 6-month follow-up visit in the first year. Two consecutive negative results of chest CT scan and serum hCG level with a minimal interval of 6 months are necessary to consider the success of the fertility-sparing treatment. From the second to the fifth year, 3-month to semi-annual monitoring of hCG and annual chest CT scan is recommended, followed by semi-annual to annual assessments of hCG onward and throughout the patient’s lifetime. From the fifth to the 10th year, a CT scan should be performed at least biennially. Additionally, an annual gynecological ultrasound scan is advised, with the option of hysteroscopy if necessary. Any persistently low serum hCG levels between annual imaging examinations, after exclusion of false positive results, are worthy of attention in the long-term of the fertility-sparing treatment. Further examinations including CT or excisional biopsy should be performed to evaluate the progression or relapse of ETT to help make a timely decision on whether to discontinue the conservative treatment.

The patient is currently 32 years old, relatively young, and has already given birth to two children. If there is a desire for further childbirth, how can a new beginning be achieved? Given the limited case reports, there is only one reference data for the fertility outcome of isolated pulmonary ETT patients who received uterine and unilateral ovary preservation therapy because of misdiagnosis with germ-cell ovarian tumor (21). In this special case, an unplanned pregnancy happened during the period of estrogen therapy for chemotherapy-relevant ovarian failure and developed into full-term vaginal delivery with normal placenta, followed by negative β-hCG. Further studies are needed to verify the effectiveness and safety of this approach. Scarce data from other successful cases of fertility-sparing management has been reported in stage I ETT patients (**Table 3**). Nine patients underwent fertility-sparing surgery, including the abdominal removal of uterine lesions (*n* = 2) (8), D&C (*n* = 1) (29), hysteroscopic removal of uterine lesions (*n* = 3) (8, 31), and laparoscopic removal of uterine lesions (*n* = 3) (4, 28, 30). Among these patients, up to follow-up date, four had no available records of subsequent gestation and one experienced an early miscarriage (8). Another two successfully gave birth through cesarean section (8, 28), one of whom had cesarean section and normal vaginal delivery about 4 and 7.5 years after the initial diagnosis, respectively (28). Two failed cases have been reported (29). In one case, segmental curettage was performed, but the disease progressed quickly and metastasized to the lungs. In another case, the patient suffered a spontaneous abortion, followed by local relapse of ETT in the uterus and cervix.

**Table 3:** Clinical features of patients managed with fertility-sparing surgery treatment of ETT in the literature.

Author	Patient No.	Age, years	Ethnicity	Gravida	Parity	Symptoms	Preceding pregnancy	Interval from last pregnancy, months	Pretreatment hCG, mIU/mL	FIGO stage	Mass features	Treatment	Overall outcome	Fertility outcome	Interval from remission to next pregnancy	Recurrence	Survival, months
Liu W et al. (8)	1	NA	Asia, Chinese	NA						I	NA	Abdominal removal of uterine lesions + multi-agent chemotherapy	Remission	One of them had term birth by CS	NA	–	NA
	2			NA													
	3	NA	Asia, Chinese	NA						I	NA	Hysteroscopic lesionectomy + multi-agent chemotherapy	Remission		NA	–	NA
	4	19	Asia, Chinese	2	1	Vaginal spotting	Induced abortion	24	100	I	2.4 × 1.6 × 1.4 cm in uterine cavity	D&C + hysteroscopic lesionectomy + 3 cycles of EMA-EP	Remission	A first-trimester miscarriage	NA	–	≥20
Tse K Y et al. (28)	5	25	Asia, Chinese	1	0	Abdominal pain	Abortion	21	NA	I	11.9 × 10.4 × 8.5 cm over the posterior uterine serosa	Laparoscopic removal of posterior uterine cyst	Remission	2 full-term births (1 CS and 1 vaginal delivery)	4 and 7.5 years	–	89
Davis M R et al. (29)	6	31	NA	1	0	VB	partial HM	24	150,000	I	<1 cm in uterus	D&C	Progressive disease (lung metastasis after 26 months)	NA	/	+	NA
	7	31	NA	0	0	None	An undocumented spontaneous abortion	NA	<2	NA (peritoneum metastasis)	<3 cm in uterus	Laparoscopic myomectomy and morcellation + EMA-EP	Stable disease (uterus and cervix recurrence after 26 months)	Spontaneous abortion	NA	+	NA
Fang FY et al. (30)	8	28	NA	1	1	Abdominal pain, heavy VB	TPY	NA	2,259–2,764	I	7.0 × 7.0 × 6.0 cm in the right cornual area of uterus	Laparoscopic lesionectomy and uterine repair	Remission	NA	/	–	NA
Zhang et al. (31)	9	42	Asia, Chinese	NA	NA	Irregular VB	HM	36	96	II	2.0 ×2.0 × 2.0 cm in the right broad ligament of uterus	Lesionectomy	Remission	NA	/	–	28

VB, vaginal bleeding; TPY, term pregnancy; HM, hydatidiform mole; CS, caesarean section.

+ positive; - negative; NA not available; / blank.

The patient has three lung metastatic lesions in total, indicating a certain risk of recurrence. Considering the shortest interval of conception from the remission year in reported cases and the optimal reproductive age for women, it might be favorable for our patient to attempt conception after the last complete remission period of at least 3 years. Once the pregnancy plan is started, the differential diagnosis between gestation and ETT relapse would be priority, which is a big challenge especially during early pregnancy. Additionally with the possibility of tumor relapse after subsequent pregnancies and deliveries, we advised our patient to perform uterine removal at the end of the third pregnancy. If relapse came before pregnancy during fertility-sparing management, uterine removal is also recommended to be supplemented in the following surgical treatment plan. Our study is limited by the small sample size and relatively short follow-up periods, and more long-term studies are needed to confirm the safety and effectiveness of this experimental treatment.

## Conclusion

ETT typically lacks specific symptoms, resulting in a complex diagnostic process. When young women experience persistent mildly elevated serum hCG levels, especially with lung lesions but no abnormalities in reproduction, ETT should be highly suspected. The treatment for isolated lung ETT may be determined individually, taking into consideration the desire for fertility, tumor behaviors, and response to therapy. For patients who wish to preserve their fertility, it is feasible to remove isolated lung lesions without removing the uterus. The fertility preservation management of ETT requires a multidisciplinary collaboration involving gynecologists, oncologists, and pathologist. Personalized treatment plans should be tailored to ensure long-term care and reliable disease surveillance. Whether this strategy makes the situation better rather than worse calls for more research to define.

## Data availability statement

The original contributions presented in the study are included in the article/supplementary material. Further inquiries can be directed to the corresponding author.

## Ethics statement

Written informed consent was obtained from the individual(s) for the publication of any potentially identifiable images or data included in this article.

## Author contributions

ZH: Conceptualization, Data curation, Formal analysis, Writing – original draft. YY: Data curation, Resources, Writing – review & editing. DW: Data curation, Visualization, Writing – original draft. NW: Writing – review & editing. LZ: Conceptualization, Project administration, Supervision, Writing – review & editing.

## References

[1] Froeling FEM Ramaswami R Papanastasopoulos P Kaur B Sebire NJ Short D . Intensified therapies improve survival and identification of novel prognostic factors for placental-site and epithelioid trophoblastic tumours. Br J Cancer. (2019) 120:587–94. doi: 10.1038/s41416-019-0402-0 PMC6461960 30792530

[2] Li JW Hu CC Shi HY Wu RJ . Extrauterine epithelioid trophoblastic tumors presenting as lung mass: A case report and literature review. Med (Baltimore). (2019) 98:e14010. doi: 10.1097/MD.0000000000014010 PMC6380824 30702558

[3] Shih IM Kurman RJ . Epithelioid trophoblastic tumor: a neoplasm distinct from choriocarcinoma and placental site trophoblastic tumor simulating carcinoma. Am J Surg Pathol. (1998) 22:1393–403. doi: 10.1097/00000478-199811000-00010 9808132

[4] Ngan HY Bender H Benedet JL Jones H Montruccoli GC Pecorelli S . Gestational trophoblastic neoplasia, FIGO 2000 staging and classification. Int J Gynaecol Obstet. (2003) 83 Suppl 1(0020-7292:175–7. doi: 10.1016/S0020-7292(03)90120-2 14763174

[5] G Deng J Gao Y Zhang J Li Liaoning Montruccoli GC Pecorelli S. Guidelines for diagnosis and treatment of tubal pregnancy in integrative medicine practice. Practice guideline REgistration for transPAREncy. 2019-08-26.

[6] Gadducci A Carinelli S Guerrieri ME Aletti GD . Placental site trophoblastic tumor and epithelioid trophoblastic tumor: Clinical and pathological features, prognostic variables and treatment strategy. Gynecol Oncol. (2019) 153:684–93. doi: 10.1016/j.ygyno.2019.03.011 31047719

[7] Frijstein MM Lok CAR van Trommel NE Ten Kate-Booij MJ Massuger L van Werkhoven E . Management and prognostic factors of epithelioid trophoblastic tumors: Results from the International Society for the Study of Trophoblastic Diseases database. BMC Cancer. (2019) 152:361–7. doi: 10.1016/j.ygyno.2018.11.015 30473257

[8] Liu W Zhou J Yang J Huang X . A multicenter retrospective study of epithelioid trophoblastic tumors to identify the outcomes, prognostic factors, and therapeutic strategies. Front Oncol. (2022) 12(2234-943X:907045. doi: 10.3389/fonc.2022.907045 35677151 PMC9169038

[9] Hsiue EH Hsu C Fau - Tseng L-H Tseng Lh Fau - Lu T-P Lu Tp Fau - Kuo K-T Kuo KT . Epithelioid trophoblastic tumor around an abdominal cesarean scar: A pathologic and molecular genetic analysis. Int J Gynecol Pathol. (2017) 36:562–7. doi: 10.1097/PGP.0000000000000366 28134666

[10] Abu-Rustum NR Yashar CM Arend R Emma B Bradley K Brooks R NCCN Clinical Practice Guidelines in Oncology: Gestational Trophoblastic Neoplasia (2024.V1). National Comprehensive Cancer Network. 2023-10-27. 10.6004/jnccn.2019.0053 31693991

[11] Kim JH Lee SK Hwang SH Kim JS Yoon G Lee YY . Extrauterine epithelioid trophoblastic tumor in hysterectomized woman. Obstet Gynecol Sci. (2017) 60:124–8. doi: 10.5468/ogs.2017.60.1.124 PMC5313356 28217684

[12] Li J Wang Y Lu B Lu W Xie X Shen Y . Gestational trophoblastic neoplasia with extrauterine metastasis but lacked uterine primary lesions: a single center experience and literature review. BMC Cancer. (2022) 22:509. doi: 10.1186/s12885-022-09620-2 35524210 PMC9077999

[13] Lei W Zhang F Zheng C Zhao C Tu S Bao Y . Metastatic epithelioid trophoblastic tumor of the lung: A case report. Med (Baltimore). (2018) 97:e0306. doi: 10.1097/MD.0000000000010306 PMC5916664 29668580

[14] Urabe SFH Miyoshi H Arihiro K Soma H Yoshihama I Mineo S . Epithelioid trophoblastic tumor of the lung. J Obstet Gynaecol Res. (2007) 33:397–401. doi: 10.1111/j.1447-0756.2007.00545.x 17578376

[15] Lewin SN Aghajanian C Moreira AL Soslow RA . Extrauterine epithelioid trophoblastic tumors presenting as primary lung carcinomas: morphologic and immunohistochemical features to resolve a diagnostic dilemma. Am J Surg Pathol. (2009) 33:1809–14. doi: 10.1097/PAS.0b013e3181b9cd67 19773636

[16] Okereke IC Chen S . Primary epithelioid trophoblastic tumor of the lung. Ann Thorac Surg. (2014) 97:1420–1. doi: 10.1016/j.athoracsur.2013.07.031 24694417

[17] Kim JY An S Jang SJ Kim HR . Extrauterine epithelioid trophoblastic tumor of lung in a 35-year-old woman. Korean J Thorac Cardiovasc Surg. (2013) 46:471–4. doi: 10.5090/kjtcs.2013.46.6.471 PMC3868698 24368977

[18] Ahn HY Hoseok I Lee CH Jung YJ Shin NR Kim KH . Pulmonary mass diagnosed as extrauterine epithelioid trophoblastic tumor. Thorac Cardiovasc Surg. (2013) 61:97–100. doi: 10.1055/s-00000085 23307273

[19] Abrão FC Sabbion RO Canzian M Fernandez A Fushida K Fernandes PM . Isolated epithelioid trophoblastic tumor mimicking non-small cell lung cancer. J Thorac Oncol. (2011) 6:966–7. doi: 10.1097/JTO.0b013e318215a214 21623270

[20] Sobecki-Rausch JWA Maniar KP Hoekstra AV Berry E Novak K Lurain JR . Surgery and platinum/etoposide-based chemotherapy for the treatment of epithelioid trophoblastic tumor. Int J Gynecol Cancer. (2018) 28:1117–22. doi: 10.1097/IGC.0000000000001278 29757875

[21] Fénichel PRC Butori C Chevallier P Poullot AG Thyss A Mouroux J . Extragestational βHCG secretion due to an isolated lung epithelioid trophoblastic tumor: microsatellite genotyping of tumoral cells confirmed their placental origin and oriented specific chemotherapy. J Clin Endocrinol Metab. (2014) 99:3515–20. doi: 10.1210/jc.2014-1460. 25029419

[22] Hamazaki S Nakamoto S Okino T Tsukayama C Mori M Taguchi K . Epithelioid trophoblastic tumor: morphological and immunohistochemical study of three lung lesions. Hum Pathol. (1999) 30:1321–7. doi: 10.1016/S0046-8177(99)90063-1 10571512

[23] Ngu S-F Ngan HYS . Surgery including fertility-sparing treatment of GTD. Best Pract Res Clin Obstetrics Gynaecol. (2021) 74:97–108. doi: 10.1016/j.bpobgyn.2020.10.005 PMC7547826 33127305

[24] Horowitz NS Goldstein DP Berkowitz RS . Placental site trophoblastic tumors and epithelioid trophoblastic tumors: Biology, natural history, and treatment modalities. Gynecol Oncol. (2017) 144:208–14. doi: 10.1016/j.ygyno.2016.10.024 27789086

[25] Mangili G Sabetta G Cioffi RA-O Rabaiotti E Candotti GA-O Pella F . Current evidence on immunotherapy for gestational trophoblastic neoplasia (GTN). . Cancers (Basel). (2022) 14:2782. doi: 10.3390/cancers14112782 35681761 PMC9179472

[26] Ghorani E Kaur B Fisher RA Short D Joneborg U Carlson JW . Pembrolizumab is effective for drug-resistant gestational trophoblastic neoplasia. Lancet. (2017) 390:2343–5. doi: 10.1016/S0140-6736(17)32894-5 29185430

[27] Bell SG Uppal S Sakala MD Sciallis AP Rolston A . An extrauterine extensively metastatic epithelioid trophoblastic tumor responsive to pembrolizumab. Gynecol Oncol Rep. (2021) 37(2352-5789:100819. doi: 10.1016/j.gore.2021.100819 34258359 PMC8258853

[28] Tse KY Chiu KWH Chan KKL Chu MMY Ngu SF Cheung ANY . A case series of five patients with pure or mixed gestational epithelioid trophoblastic tumors and a literature review on mixed tumors. Am J Clin Pathol. (2018) 150:318–32. doi: 10.1093/ajcp/aqy039 29897391

[29] Davis MR Howitt BE Quade BJ Crum CP Horowitz NS Goldstein DP . Epithelioid trophoblastic tumor: A single institution case series at the New England Trophoblastic Disease Center. Gynecol Oncol. (2015) 137:456–61. doi: 10.1016/j.ygyno.2015.03.006 25773203

[30] Fang FY Lai CR Yang MJ Huang BS Chen CY Li YT . Diagnostic challenges in cornual epithelioid trophoblastic tumor. Taiwan J Obstet Gynecol. (2014) 53:235–8. doi: 10.1016/j.tjog.2014.03.002 25017275

[31] Zhang Y Zhang SA-O Huang W Chen T Yuan H Zhang Y . Intermediate trophoblastic tumor: the clinical analysis of 62 cases and prognostic factors. Arch Gynecol Obstet. (2019) 299:1353–64. doi: 10.1007/s00404-018-05037-0 30607597

